# Reprogramming chick RPE progeny cells to differentiate towards retinal neurons by *ash1*

**Published:** 2008-12-12

**Authors:** Weiming Mao, Run-Tao Yan, Shu-Zhen Wang

**Affiliations:** Department of Ophthalmology, University of Alabama at Birmingham, Birmingham, AL

## Abstract

**Purpose:**

Harnessing a cell culture of retinal pigment epithelium (RPE) to give rise to retinal neurons may offer a source of developing neurons for cell-replacement studies. This study explores the possibility of reprogramming RPE progeny cells to differentiate toward retinal neurons with achaete-scute homolog 1 (*ash1*), a proneural gene that is expressed in progenitor cells in the developing retina and promotes amacrine cell production when overexpressed in the chick retina.

**Methods:**

Replication Competent Avian Splice (RCAS) retrovirus was used to drive the ectopic expression of *ash1* in cell cultures of dissociated RPE isolated from day 6 chick embryos. RCAS expressing green fluorescent protein (RCAS-GFP) was used as control. The cultures were examined for de novo generation of neuron-like cells by molecular, cellular, and physiologic criteria.

**Results:**

In control cultures infected with RCAS-GFP, RPE cells appeared cobblestone-like and often darkly pigmented. In cultures infected with RCAS-*ash1*, however, cells remained de-pigmented and frequently formed clusters. Further examination at the morphological and molecular levels showed the development of elaborate processes characteristic of neurons and the expression of genes/markers that identify different types of retinal neurons. The most prevalently expressed neural marker was calretinin, which in the chick retina identifies amacrine, ganglion, and horizontal cells. As an assay for functional maturation, the reprogrammed cells were analyzed for the presence of functional, ionotropic glutamate receptors that lead to a rise in the cytosolic free calcium (Ca^2+^) concentration. Calcium imaging showed that reprogrammed cells responded to glutamate and N-methyl-D-aspartate (NMDA) by increasing their Ca^2+^ concentrations, which, after reaching a peak level, returned to the basal level. The response curves of reprogrammed cells resembled those of cultured retinal neurons.

**Conclusions:**

These results suggest that RPE progeny cells can be reprogrammed by *ash1* to develop molecular, morphological, and physiologic properties that are characteristic of retinal neurons.

## Introduction

The vertebrate retina contains five major types of neurons: photoreceptor, horizontal, bipolar, amacrine, and ganglion. Visual signals are initiated by photoreceptors, modulated by horizontal, bipolar, and amacrine cells, and transmitted to the brain through the axons of ganglion cells. Retinal neurons, like other neurons in the central nervous system, are terminally differentiated and do not reenter the cell cycle for proliferation. Thus, degenerated retinal neurons are not automatically replaced. In the teleost retina, both progenitor/stem cells at the ciliary marginal zone and Müller glia in the retina can give rise to new retinal cells in response to injury [1-5]. Chick Müller glia have also been shown to exhibit some properties of retinal stem cells [6]. Nonetheless, such an inborn stem-cell-based regeneration mechanism seems elusive in the retina of higher vertebrates. As a result, attention in recent years has been directed toward inducing retinal neurogenesis through programming or reprogramming the differentiation of cells that can be propagated in large numbers in vitro, including embryonic stem cells and adult stem cells of the brain, retina, or bone marrow [7-11].

Unlike retinal neurons, retinal pigment epithelial (RPE) cells from many species, including human, can reenter the cell cycle. More importantly, their progeny are able to differentiate into cell types other than RPE [12], suggesting the possibility of RPE serving as retinal stem cells [13]. The idea of using RPE cell cultures as a novel source of developing retinal neurons has been experimentally explored. Most of the published reports examined genes/factors that are involved in the production of photoreceptors [14-17] or ganglion cells [18,19].

A large number of studies have shown that *ash1*, a vertebrate homolog of the Drosophila proneural gene *achaete*-*scute*, exhibits proneural activity during the development of both the central and peripheral nervous systems [20-28], where *ash1* promotes neurogenesis against gliogenesis [29-32]. *Ash1* encodes a transcription factor of the basic helix–loop–helix family. In the retina, expression of *ash1* is restricted to progenitor cells [29,33]. In the mouse, *ash1* is required in the production of late-born neurons, including rod photoreceptors and bipolar cells [29]. In *ash1*;*ath3* double knockouts, the production of bipolar cells is virtually abolished [34]. However, misexpression of *ash1* or *ath3* alone does not promote bipolar cell genesis, but co-misexpression of *ash1*, *ath3*, and *chx10* increases bipolar cell number [35]. Transgenic expression of *ash1* in mouse RPE initiates retinal neurogenesis in the RPE cell layer [36]. In the chick, the temporal and spatial pattern of *ash1* expression coincides with amacrine cell genesis [33], and overexpression of *ash1* expands the amacrine population [37].

Making use of RPE cells’ plasticity and the suggested roles of *ash1* in the production of retinal progenitor cells and amacrine cells, we studied the possibility of coaxing cultured RPE cells with *ash1* into differentiating along retinal neural pathways. Our experiments demonstrate neural differentiation at the molecular, morphological, and physiologic levels in the otherwise non-neural RPE cell cultures infected with a retrovirus expressing *ash1*.

## Methods

### Chick embryos

Fertilized, pathogen-free White Leghorn chicken eggs were purchased from Spafas (Preston, CT) and incubated in a Petersime egg incubator (Gettysburg, OH). The care and use of animals adhered to the procedures and policies comparable to those published by the USA Public Health Service (Public Health Service Police on Humane Care and Use of Animals) and set by the Institutional Animal Use and Care Committee at the University of Alabama at Birmingham.

### Replication Competent Avian Splice retroviruses

Replication Competent Avian Splice (RCAS) retrovirus [38] expressing *ash1* (RCAS-*ash1*) was generated as described [37]. During cloning process, a recombinant (rb) form of Ash1, Ash1ΔC_rb_, was generated. It lacked the C-terminal 33 amino acids of the wild-type Ash1 and contained (at the C-terminus) 19 extra residues derived from the shuttle vector Cla12Nco. RCAS-*ash1ΔC_rb_* was included in the study to determine whether the C-terminus of Ash1 was dispensable for its function. All recombinant RCAS constructs were sequenced for verification and for ruling out possible changes including frameshift or nonsense mutations. Sequencing was performed at the DNA Sequencing Service of the Genomics Core Facility at the University of Alabama at Birmingham. RCAS-green fluorescent protein (GFP) was produced as previously described [14].

### RPE cell culture

E6 chick eyes were enucleated, and tissues outside the PRE were removed. A circular incision in the ciliary region was made to get rid of the anterior portion of the eye, producing eye cup consisting of the RPE, the retina, and the vitreous. The RPE was then peeled off from the neural retina. RPE tissues were dissected from day 6 chick embryos (E6) as previously described [14]. Cells in RPE tissues were dissociated by trypsin/EDTA and gentle trituration. Fully dissociated RPE cells were resuspended in Gibco medium 199 (Invitrogen, Carlsbad, CA) plus 10% fetal bovine serum. The cells were then seeded at low density (approximately 1×10^5^ cells) in a 35 mm dish and cultured at 37 °C under 5% CO_2_ with Knockout Dulbecco's Modified Eagle Medium (D-MEM) supplemented with 20% serum replacement (Invitrogen). After 3–4 days in culture, when 50% confluency was reached, 10–15 μl of retrovirus stock (2–5×10^8^ pfu/ml) was added to each 35 mm dish. After an additional 8–12 days in culture, cells were directly subjected to Ca^2+^ imaging or fixed with ice-cold 4% paraformaldehyde in phosphate-buffered saline (PBS) for analyses with immunocytochemistry and in situ hybridization.

To facilitate visualizing the morphologies of reprogrammed cells, we transfected RPE cell cultures 6 days after the administration of RCAS-*ash1ΔC_rb_*. Transfection was done with recombinant DNA of adeno-associated virus expressing GFP (AAV-GFP) driven by a chimerical promoter of Cytomegalovirus (CMV)-actin-β globin. Cellular morphologies were imaged with a blue filter under a 20X or 40X objective attached to a Nikon TE300 (Melville, NY) inverted microscope 2–4 days after AAV-GFP DNA transfection.

The calcium indicator fluo-4 AM [39] (Molecular Probes, Eugene, OR) was also used to visualize the morphologies of reprogrammed cells. Cells in RPE cell cultures infected with RCAS-*ash1* or RCAS were incubated with fluo-4 AM as described in detail late under “Calcium imaging” [17]. Cellular morphologies were imaged under a 40X objective attached to a Nikon TE300 inverted microscope with a blue filter.

### Retinal cell culture

Retinas were dissected from day 16 chick embryos. Retinal cells were dissociated with 0.05% trypsin and 0.53 mM EDTA (Invitrogen) and mechanical trituration. Dissociated retinal cells were cultured in polyornithine-treated 35 mm dishes with medium 199 (Invitrogen) plus 10% fetal bovine serum at 37 °C under 5% CO_2_ for 5 days. Culture medium was changed every other day, and the cells were subjected to Ca^2+^ imaging in the same way as those in RPE cell cultures.

### Immunocytochemistry

The following monoclonal antibodies were obtained from the Developmental Studies Hybridoma Bank (Iowa University, Iowa City, IA): anti-AP2 (3B5; 1:50; developed by Dr. T. Williams, Yale University, New Haven, CT); anti-islet-1 (1:100; developed by Dr. T. Jessell, Columbia University, New York, NY), anti-LIM (4F2; 1:50; developed by Dr. T. Jessell, Columbia University, New York, NY), anti-vimentin (H5; 1:500; developed by Dr. J. Sanes, Harvard University, Cambridge, MA), and anti-visinin (7G4; 1:500; developed by Dr. C. Cepko, Harvard University, Cambridge, MA). Antibodies obtained from commercial sources included antiviral protein p27 (1:500; Spafas, Preston, CT), polyclonal antibody against calretinin (1:500; Chemicon, Temecula, CA), polyclonal antibody against red opsin (1:200; Chemicon), polyclonal antibody against AP2α (1:200; Santa Cruz Biotechnology, Santa Cruz, CA), and monoclonal antibody against MAP2 (1:200; Sigma, St. Louis, MO). Monoclonal antibody RA4 (1:1,000 dilution) was a gift from Dr. Steven McLoon (University of Minnesota, Minneapolis, MN). Standard procedures for immunocytochemistry were followed.

The number of immunopositive cells was counted under a Nikon TE300 microscope (Melville, NY). In each experiment, 3 dishes were assigned for each marker. From each dish, the number of immunopositive cells was scored from 10 view areas under a 20X objective, and the total number of immunopositive cells per 35 mm dish was calculated by multiplying the average per view area by 200 (the total number of view areas per dish).

### In situ hybridization

Briefly, cells in the culture were fixed with ice-cold 4% paraformaldehyde for 30 min before being permealized with 0.25% triton X-100 for 30 min. Hybridization was carried with 40–80 ng of RNA probes overnight at 48–55 °C. In situ hybridization was used to detect the expression of interphotoreceptor retinal binding protein (*IRBP*), GAD67 (GenBank 4103977), *ath3*, and *chx10*, as previously described [15,40].

### Calcium imaging

Calcium imaging was used to study the physiologic responses of reprogrammed cells to neurotransmitters, as described by Lamba et al. [11] with minor modifications. Cells in the 35 mm culture dish (either of RPE cell culture or of retinal cell culture) were washed and incubated with medium 199 for 1 h at 37 °C. Then cells were washed with a magnesium-free bath solution that contained 5.3 mM KCl, 115.0 mM NaCl, 3.1 mM CaCl_2_, 5.6 mM glucose, and 3.0 mM HEPES, pH 7.2. The bath solution was adapted from that given in Sosa and Gleason [41]. Next, cells were incubated with fluo-4 AM as described [17] in 1 ml of bath solution for 15 min at room temperature. After a brief rinsing and then incubation with the bath solution for 30 min at room temperature, the culture dish was placed under an Olympus BX60 microscope with constant perfusion with the bath solution at a rate of 1 ml per minute at room temperature. Fluorescence images were captured sequentially without time interval with 400 ms-exposure for each image. Captured images were analyzed with WCFI ImageJ software. Briefly, a region of interest covering the soma of a cell was selected in stacked images. A region of the same shape and area was also selected from the background for background subtraction. Changes in fluorescence intensity were expressed as percentage of ΔF/Fo, where F is the averaged integrated optic density (IOD) of an image, Fo is the average of IOD the images before glutamate application, and ΔF=F-Fo. Approximately 3–5 μl of 1 mM glutamate in the bath solution, or 1 mM N-methyl-D-aspartate (NMDA) and 1 mM glycine, was puffed onto the area of interest within 500 ms through a micropipette driven by a PV800 Pneumatic Picopump (World Precision Instruments, Sarasota, FL). The recording chamber was under constant perfusion (around 1 ml/minute) at room temperature. ΔF/Fo was plotted against time using Origin Pro7 (OriginLab, Northampton, MA).

## Results

### Altered appearance of RPE cell culture infected with RCAS-*ash1*

**Figure 1:**
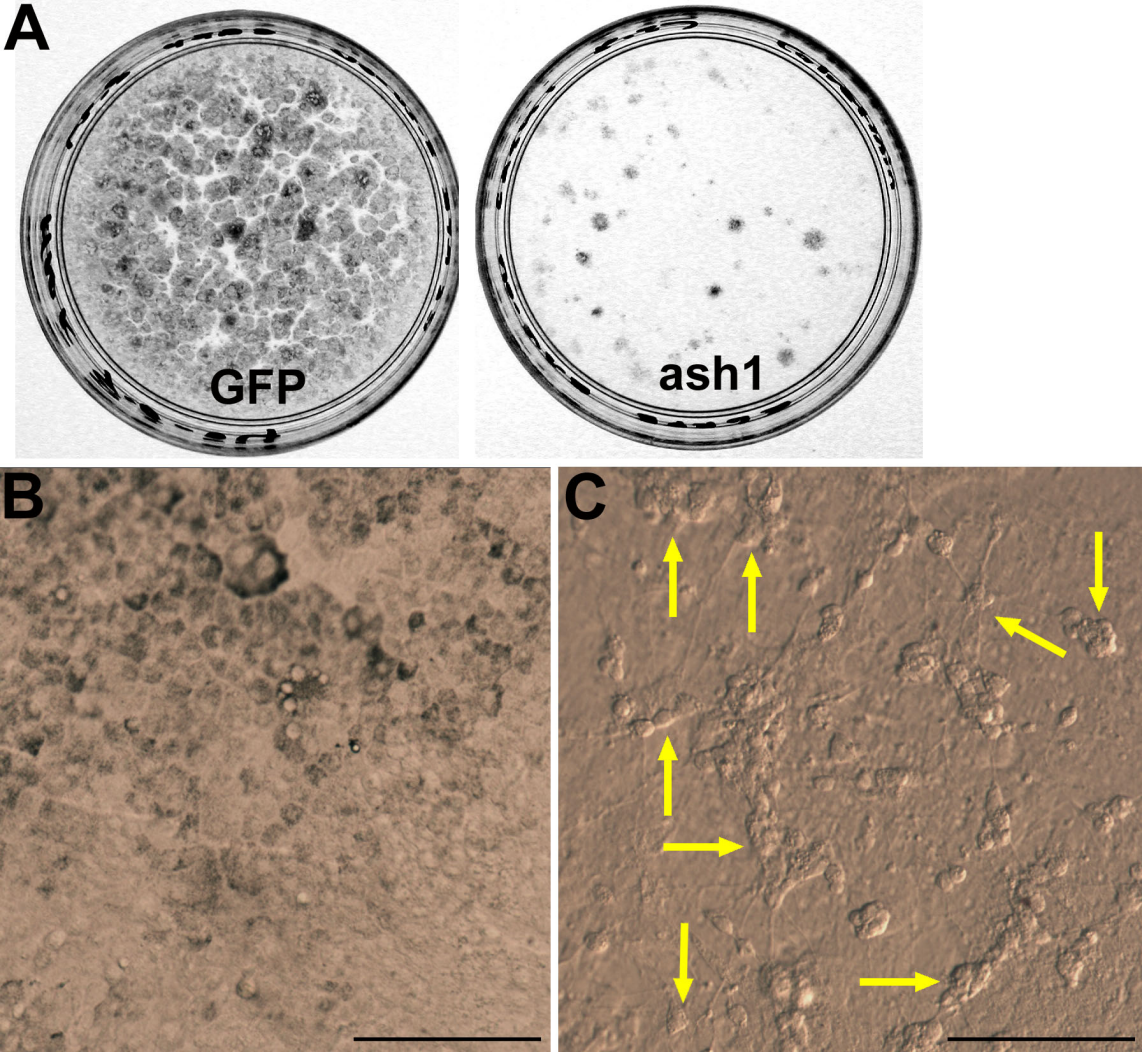
The appearance of RPE cell culture infected with RCAS-*ash1* differed from that of the control. **A:** The control retinal pigment epithelium (RPE) cell culture infected with Replication Competent Avian Splice (RCAS)-green fluorescent protein (GFP; left) showed dark pigmentation, but the experimental culture infected with RCAS-*achaete-scute homolog 1* (*ash1;* right) remained un-pigmented, except at a few places. **B:** The control culture displayed a monolayer-appearance. **C:** The experimental culture contained clusters or aggregates of cells. Arrows point to cell clusters. Scale bars represents 100 μm.

Dissociated RPE cells (seeded at a density of approximately 1×10^5^ cells in a 35 mm dish) lose their dark pigment during the initial days in culture, but they become repigmented as the culture approaches confluency [42]. Infection with RCAS-GFP has little or no effect on the repigmentation of the culture (Figure 1A,B; [42]). Infection with RCAS-*ash1*, however, prevented the RPE culture from repigmentation, except at places where patches of pigmented cells were present (Figure 1A).

In addition, cultures infected with RCAS-*ash1* also contained cell clusters noticeable under a microscope with phase contrast or Hoffman modulation optics (Figure 1C). The clusters of cells were irregularly shaped and contained cells with a round cell body and often with processes, reminiscent of neural clusters. No such cell clusters were present in the control culture, in which hexagonal cells were organized in a cobblestone fashion (Figure 1B).

### Induction of neural genes by *ash1*

To determine whether *ash1* reprogrammed RPE progeny cells to differentiate toward retinal neurons, RPE cell cultures infected with RCAS-*ash1* were examined for the expression of genes/markers that are normally expressed in various types of retinal neurons. These markers included calretinin, expressed in horizontal and ganglion cells and some amacrine cells [43,44]; general neural marker MAP2 present in all retinal neurons except bipolar cells [45]; ganglion cell markers RA4 [46] and Islet-1 [47], which also labels bipolar cells at late developmental stages, amacrine markers AP2α [48] and GAD67 [49]; bipolar cell markers *ath3* and *chx10* [50]; horizontal cell marker LIM [51]; photoreceptor markers red opsin, IRBP [52,53], and visinin [54], whose immunoreactivity is also detected weakly in developing amacrine cells in the chick retina [55,56].

**Figure 2:**
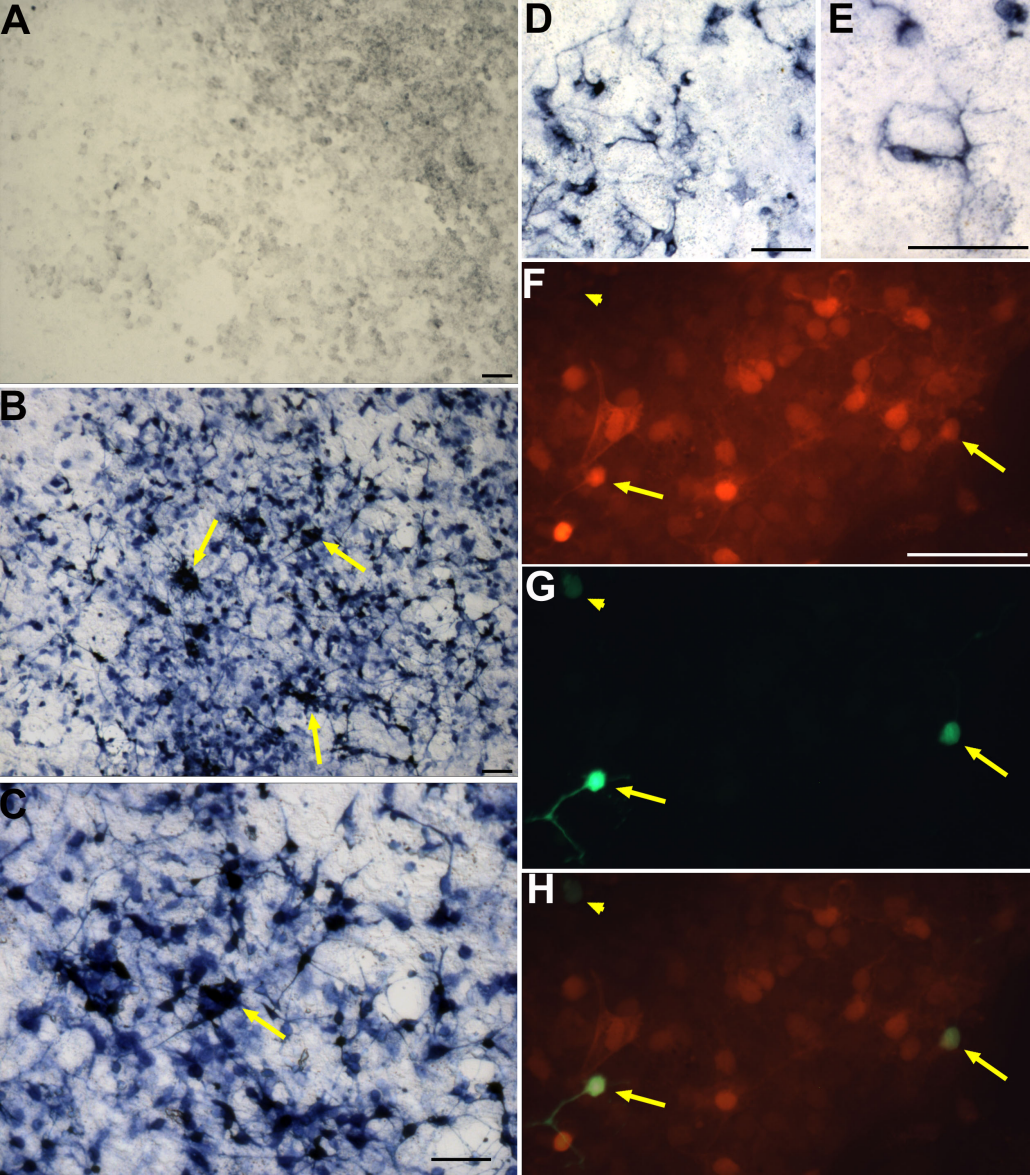
Neural markers were detected in RPE cell cultures infected with RCAS-*ash1* (or Replication Competent Avian Splice (RCAS)-*ash1*ΔC_rb_) with immunocytochemistry. **A:** Immunostaining for calretinin showed no positive cells in a control culture infected by RCAS-green fluorescent protein (GFP). **B:** A large of number of calretinin+ cells were present in a culture infected by RCAS-*ash1*. **C** is a higher magnification view of a section in **B**. Arrows point to clusters of calretinin^+^ cells. **D:** MAP2^+^ cells were present in a culture infected by RCAS-*ash1*ΔC_rb_. **E:** MAP2^+^ cells in RCAS-*ash1*ΔC_rb_-infected culture displayed neuron-like morphologies. **F-H:** Double-labeling for calretinin (**F**) and visinin (**G**) showed that a small number of calretinin^+^ cells were also visinin^+^. **H** is a merge of **F** and **G**. Arrowheads in **F**, **G**, and **H** each point to a visinin^+^ cell that lacked calretinin immunostaining. Scale bars represents 50 μm.

Overall, calretinin was the most expressed among the markers tested. While infection by RCAS-GFP did not induce calretinin expression (Figure 2A), numerous calretinin^+^ cells were detected in cultures infected with RCAS-*ash1* (Figure 2B,C). Because calretinin^+^ cells were frequently localized in clusters (Figure 2B,C, arrows), calculating their exact number was impractical; nonetheless, we estimate that as many as 20% of the cells present in the culture were calretinin^+^ (>30,000 in a 35 mm dish; Table 1). Long, thin processes typical of neurons emanated from and webbed among the calretinin*^+^* cells. Neuron-like processes were also observed on MAP2^+^ cells from RPE cultures infected with RCAS-*ash1ΔC_rb_* (Figure 2D,E).

**Table 1:** Retinal neural markers were expressed in RPE cell cultures infected with RCAS-*ash1* or RCAS-*ash1ΔC_rb_*

**Marker**	**Specificity**	**Prevalence^1^**
Calretinin	Gc, Am, Hz	++++^2^
RA4	Gc	+++
Islet-1	Gc, Bi	-^3^
GAD67	Am	++
AP2α	Am, Hz	-
Ath3	Bi	-
Chx10	Bi	-
LIM	Hz	-^3^
Visinin	Pr	++++
IRBP	Pr	+++
Red opsin	Pr	+
MAP2	all except Bi	+++^3^

^1^Shown are estimates of the numbers of cells positive for each of the markers in a 35-mm dish. ^2^Each “+” represents a log unit in the number of positive cells, i.e.; + denotes in tens; ++++ in tens of thousand; and “-” means negative. ^3^Data were from cultures infected with RCAS-*ash1ΔC_rb_*. Abbreviations: Gc represents ganglion cells; Am represents amacrine; Bi represents bipolar; Hz represents horizontal; Pr represents photoreceptor.

Immunocytochemistry and in situ hybridization of RPE cell cultures infected with RCAS-*ash1* showed the following: a substantial numbers of cells immunopositive for RA4 (>4,000 in a 35 mm dish; Table 1) or visinin (>11,000; Figure 2G); a small number of cells expressing IRBP (>1,000); a few expressing GAD67 (>100) or red opsin (>90); and none positive for AP2α, *ath3*, or *chx10* (Table 1). None of the genes/markers were expressed in the control infected with RCAS-GFP (Figure 2A) [14,16], with the exception of RA4, which typically is detected in hundreds of cells in the control [14]. We also examined the expression of neural markers in RPE cell cultures infected with RCAS-*ash1ΔC_rb_*, which expressed a truncated Ash1 lacking the C-terminal sequence and with a short sequence acquired from the cloning vector (see Methods). Chick embryos infected with RCAS-*ash1ΔC_rb_* exhibited all, but more pronounced, phenotypes observed with RCAS-*ash1* [37]. In RPE cell culture, infection with RCAS-*ash1ΔC_rb_* produced similar results as infection with RCAS-*ash1*, providing further support to the C-terminal sequence being nonessential to Ash1’s function [37].

The prevalence of calretinin^+^ cells along with *ash1* promoting amacrine overexpression in the retina [37] raised a question of whether some of the visinin^+^ cells were amacrine-like, as differentiating amacrine cells in the retina transiently express of photoreceptor markers [55,56]. Double-labeling showed the presence of visinin^+^/calretinin^+^ cells (Figure 2F-H, arrows). Double-labeled cells accounted for an estimated 75% of visinin^+^ cells (in 5 view areas under a 40X objective) and 5%–10% of calretinin^+^ cells (in the same areas).

### Morphologies of reprogrammed cells

**Figure 3:**
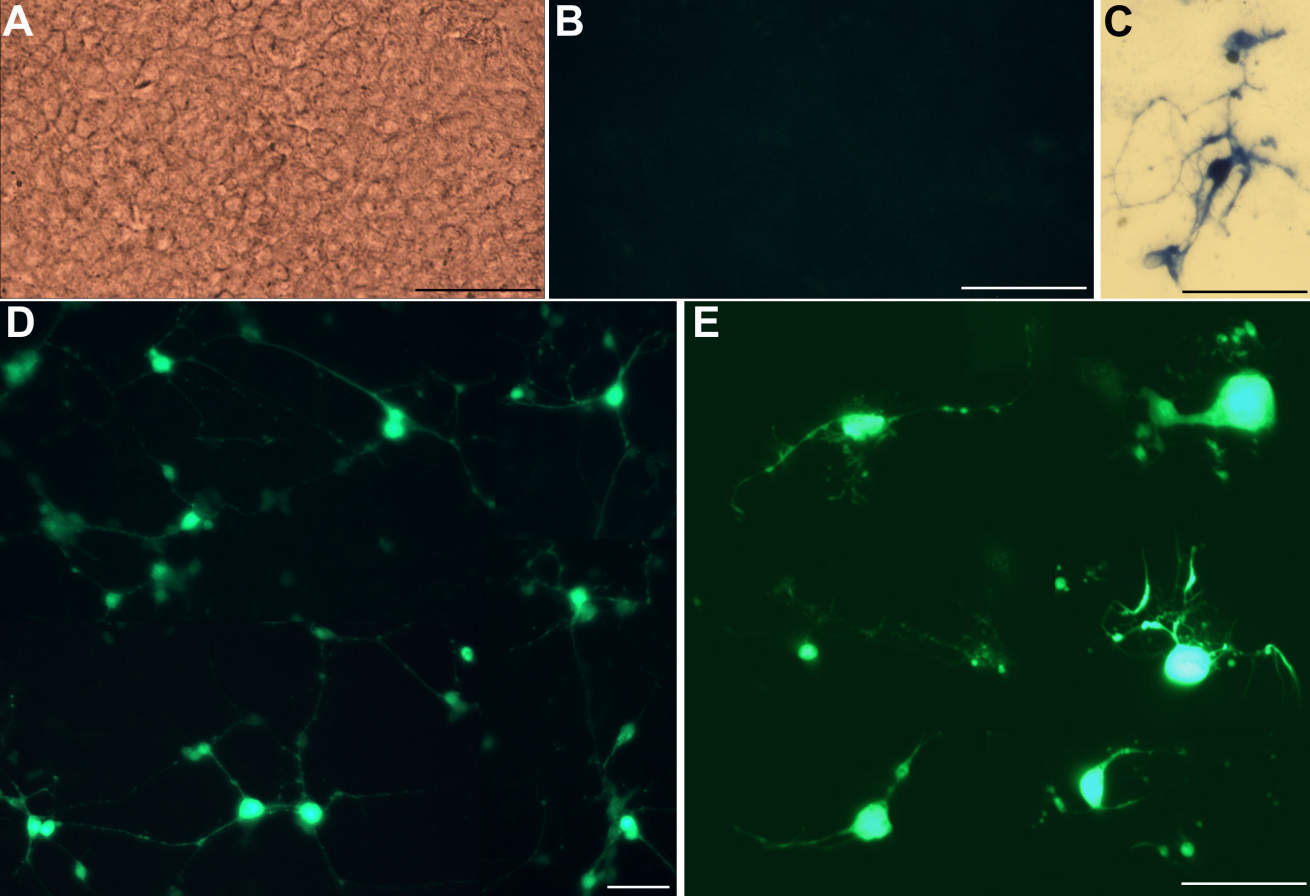
Neuron-like morphologies were formed by reprogrammed cells in RPE cell cultures infected with RCAS-*ash1* (or RCAS-*ash1*ΔC_rb_). **A:** Under bright-field, a control retinal pigment epithelium (RPE) cell culture infected with RCAS contained cells densely organized into a mono-layer. **B:** After fluo-4 AM labeling, these cells were invisible with epifluorescence, due to their low Ca^2+^ levels. **C:** A calretinin^+^ cell in an RPE cell culture infected with Replication Competent Avian Splice (RCAS)-*achaete-scute homolog 1* (*ash1)* exhibited elaborate cellular processes, reminiscent of neural processes. **D:** Reprogrammed cells in a RPE cell cultures infected with RCAS-*ash1* displayed neuron-like morphologies, as revealed with fluo-4 AM labeling. **E:** Reprogrammed cells in RCAS-*ash1*ΔC_rb_-infected culture also displayed neuron-like morphologies, as revealed with transfection of with AAV-GFP DNA. Scale bars represents 50 μm.

The calcium indicator fluo-4 AM selectively identifies reprogrammed, neuron-like cells in an RPE cell culture transduced with proneural gene *neuroD* [17]. Thus, we used fluo-4 AM coupled with fluorescence photomicrography to directly visualize the cellular morphologies of reprogrammed cells in RPE cultures infected with RCAS-*ash1*. Cells in the control cultures infected with RCAS were “invisible” under our experimental conditions (Figure 3A,B), as their intracellular Ca^2+^ concentrations were below the level of detection with a 2 s exposure in image-capture. Under the same experimental conditions, reprogrammed cultures (i.e., infected with RCAS-*ash1*) contained a large number of fluoresced cells (~15%), with the rest of the cells (presumably not being reprogrammed) remaining “invisible.” Like individual, calretinin^+^ cells with adornment by elaborate processes (Figure 3C), most of the fluoresced cells emanated multiple processes from their soma (Figure 3D), a morphological characteristic of horizontal, amacrine, or ganglion cells of the retina.

The morphology of reprogrammed cells was also examined with AAV-GFP DNA transduction. As shown in Figure 3E, reprogrammed cells in an RPE culture infected with RCAS-*ash1ΔC_rb_* displayed cellular processes remarkably complex and closely resembling those possessed by retinal horizontal, amacrine, or ganglion cells.

**Figure 4:**
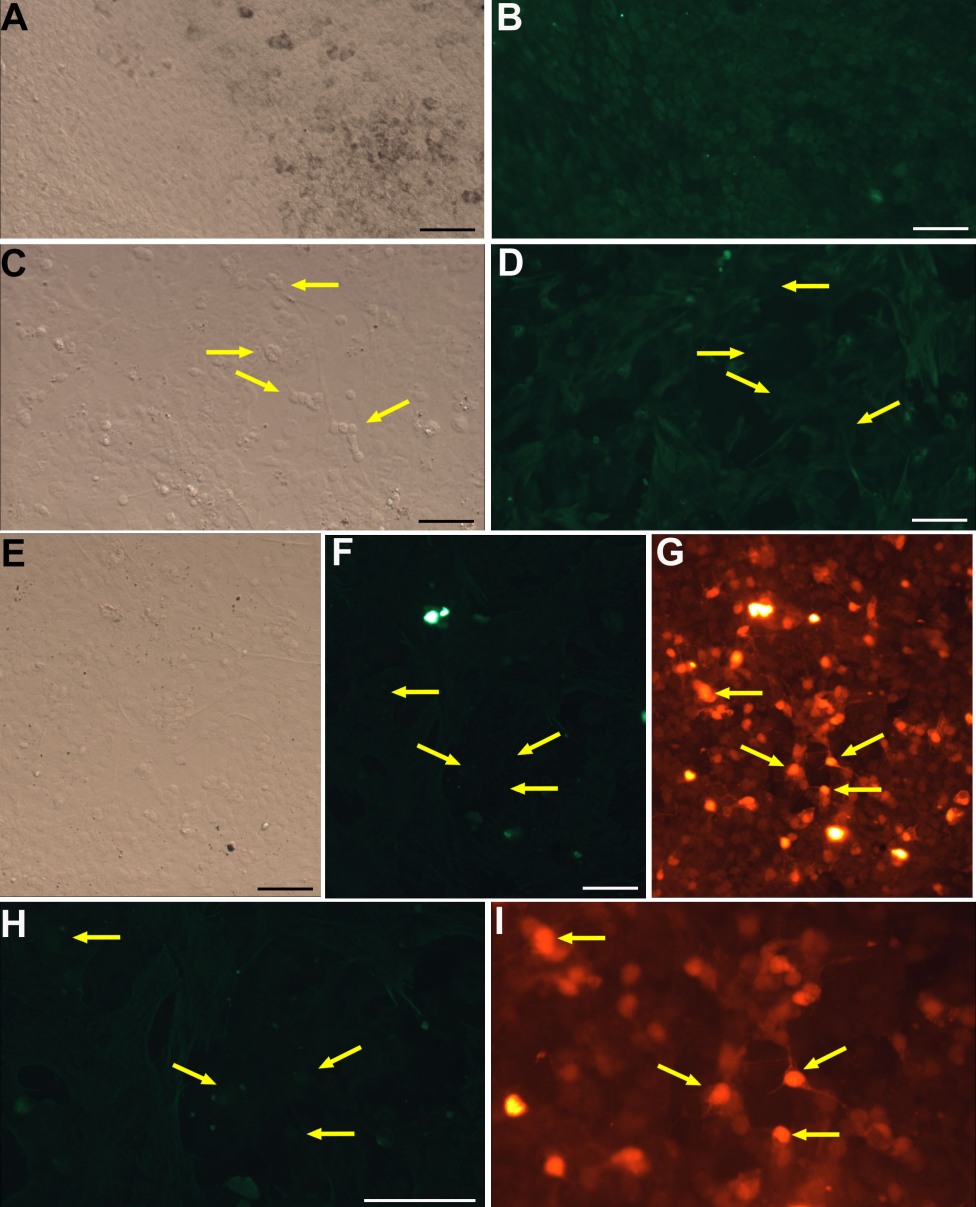
Reprogrammed cultures lacked vimentin^+^ cells that were Müller glia-like. **A, B:** Immunostaining for vimentin (**B**) showed no cells that displayed spindle-like processes (i.e., Müller glia-like) in the control culture infected with Replication Competent Avian Splice (RCAS)-green fluorescent protein (GFP), and **A** is a bright-field view of the culture. **C, D:** Reprogrammed cultures (infected with RCAS-*ash1*) also lacked vimentin^+^ cells with spindle-like processes (**D**); **C** is a bright-field view of the culture. Arrows point to cells with compact cell bodies discernible under Hoffman modulation optics. **E-G:** A lack of vimentin^+^ cells that displayed spindle-like processes in reprogrammed culture was not due to a lack of reprogramming, because double-labeling showed that no vimentin^+^ cells spindle-like processes were detected (**F**) at places where a large number of calretinin^+^ cells were present (**G**); **E** is a bright-field view of the culture. Arrows point to calretinin^+^ cells. **H** and **I** are higher magnifications of **F** and **G**, respectively. Scale bars represents 50 μm.

Because Műller glia exhibit spindle-like processes that may appear similar to neural process and its Ca^2+^ concentration may be sufficiently high to be visible with fluo-4 AM, the RPE cell cultures were examined for the presence of Müller glia-like cells by immunostaining for vimentin, which is detected in Müller glial processes [57]. Because cultured RPE cells also express vimentin and other commonly used Műller glial markers [58,59], vimentin^+^ cells with spindle-like processes were the observation target. In an RPE culture infected with RCAS-GFP, antivimentin staining was weak and observed in essentially all cells with morphologies of culture RPE cells (Figure 4A,B). Similar to the control, cultures infected with RCAS-*ash1* contained vimentin+ cells that exhibited morphologies typical of culture RPE cells, but lacked vimentin^+^ cells that displayed spindle-like processes (Figure 4C,D). No particular antivimentin immunostaining was observed at places occupied by cells with compacted cell bodies (Figure 4C,D, arrows). Double-labeling for vimentin and calretinin showed the absence of Műller glia-like, vimentin^+^ cells (Figure 4E,F,H) in a culture containing reprogrammed cells that expressed calretinin (Figure 4G,I). The absence of Müller glia-like, vimentin^+^ cells is consistent with published reports that *ash1* favors neurogenesis against gliogenesis [29-32].

### Response to glutamate and NMDA

The molecular and morphological resemblance of the reprogrammed cells to retinal neurons prompted a further analysis of whether the reprogrammed cells developed physiologic properties typical of retinal neurons. The cells were assayed for functional glutamate receptors. Glutamate is a common neurotransmitter in the retina, and its receptors are either ionotropic or metabotropic [60-62]. Ionotropic receptors include NMDA and non-NMDA receptors, varying in their selective agonists. NMDA receptors are permeable to Ca^2+^, and their activation results in an increase in intracellular Ca^2+^ concentration [62]. By comparison, most of the metabotropic receptors cause a decrease in intracellular Ca^2+^ concentration when they are activated [62].

**Figure 5:**
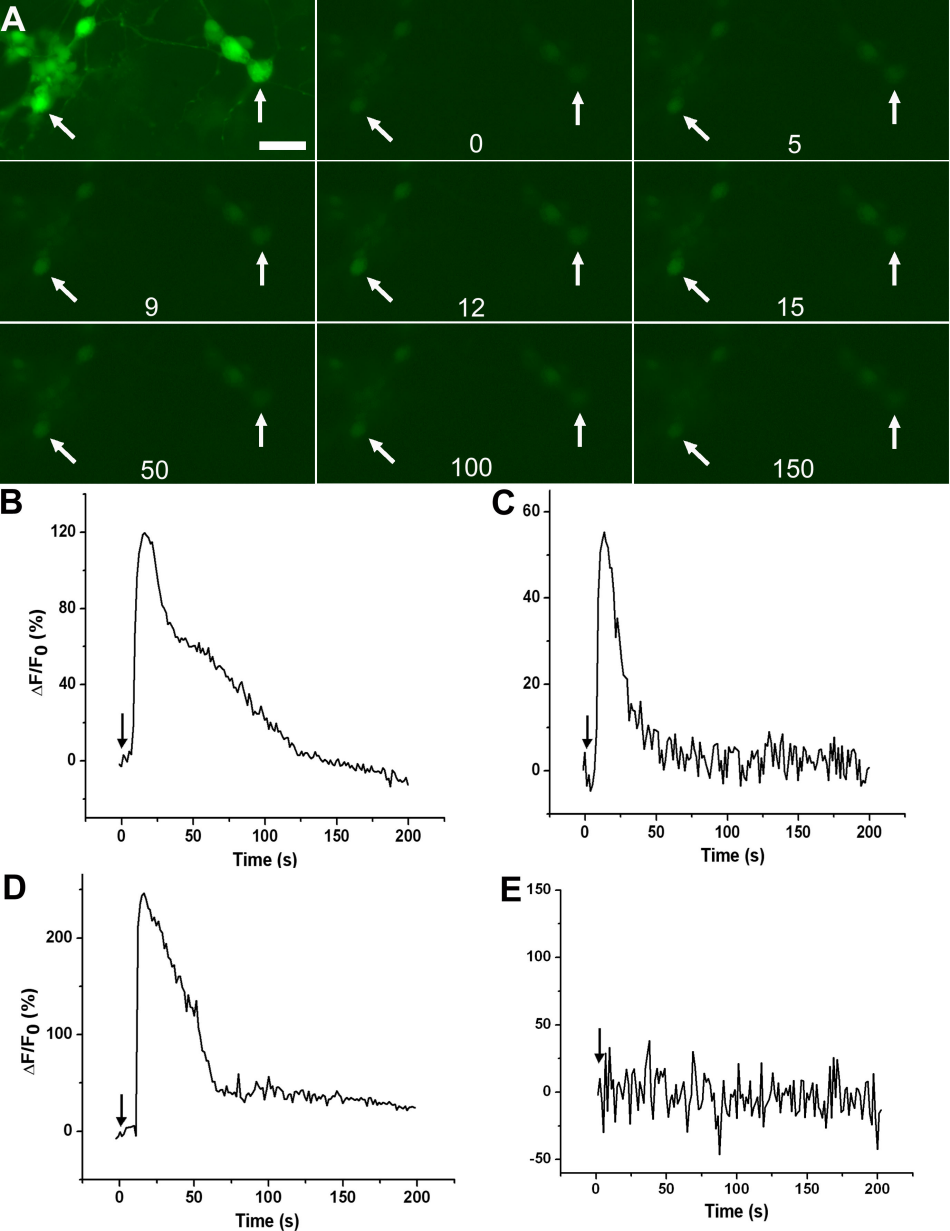
Reprogrammed cells responded to NDMA+glycine. **A:** Montage of fluorescence micrographs is shown to illustrate responses by two reprogrammed cells (arrows) in an retinal pigment epithelium (RPE) cell culture infected with RCAS-*ash1*. The image in the first panel was captured with longer exposure time for a clear view of the cells. Time (in seconds) after the application of the neurotransmitter is shown at the bottom of each panel. **B, C:** The ΔF/Fo of each of the two cells identified in **A** was plotted against time (in seconds) to produce a response curve. **D:** The ΔF/Fo a retinal cell in an E16 chick retinal cell culture was plotted against time to produce a response curve as a reference for the reprogrammed cells. **E:** The ΔF/Fo of a cell in the control RPE culture infected with RCAS was plotted against time to produce a response curve as a negative control. While no response was observed with RPE cells, reprogrammed cells responded to NDMA+glycine by transiently increasing Ca^2+^ concentrations, similar to the responses by retinal cells. The arrow in **B-E** points to the time at which the neurotransmitter was applied. Scale bars represents 50 μm.

The development of functional glutamate receptors was assayed by examining Ca^2+^ concentration before and after the administration of glutamate or NDMA along with glycine, which acts as a co-agonist of the NMDA receptor and potentiates the NMDA-triggered response [11,63]. Ca^2+^ concentration was monitored using fluo-4 AM coupled with fluorescence microscopy. We found that reprogrammed cells, particularly those with neural morphologies, responded to the administration of NMDA and glycine by increasing their Ca^2+^ concentrations (Figure 5A). Increases in fluorescence intensity were detected a few seconds after the application of the neurotransmitters, indicative of a Ca^2+^ influx through calcium channels upon receptor activation. Fluorescence intensity peaked usually within15 s, quickly declined, and eventually reached pre-activation level (Figure 5). The response was similar to that observed with retinal cells in culture. When the changes in fluorescence intensity (as ΔF/Fo) were plotted over time, the responses to the neurotransmitters by reprogrammed cells (Figure 5B,C) resembling the responses by retinal neurons (Figure 5D) was evident. Notably, the degree of response by different cells varied (Figure 5B,C), likely due to varied extents of neural differentiation and varied physiologic conditions. Additionally, approximately 20% of the 86 cells subjected to the analysis showed responses. Cells in the control culture (infected with RCAS) showed no response (Figure 5E).

**Figure 6:**
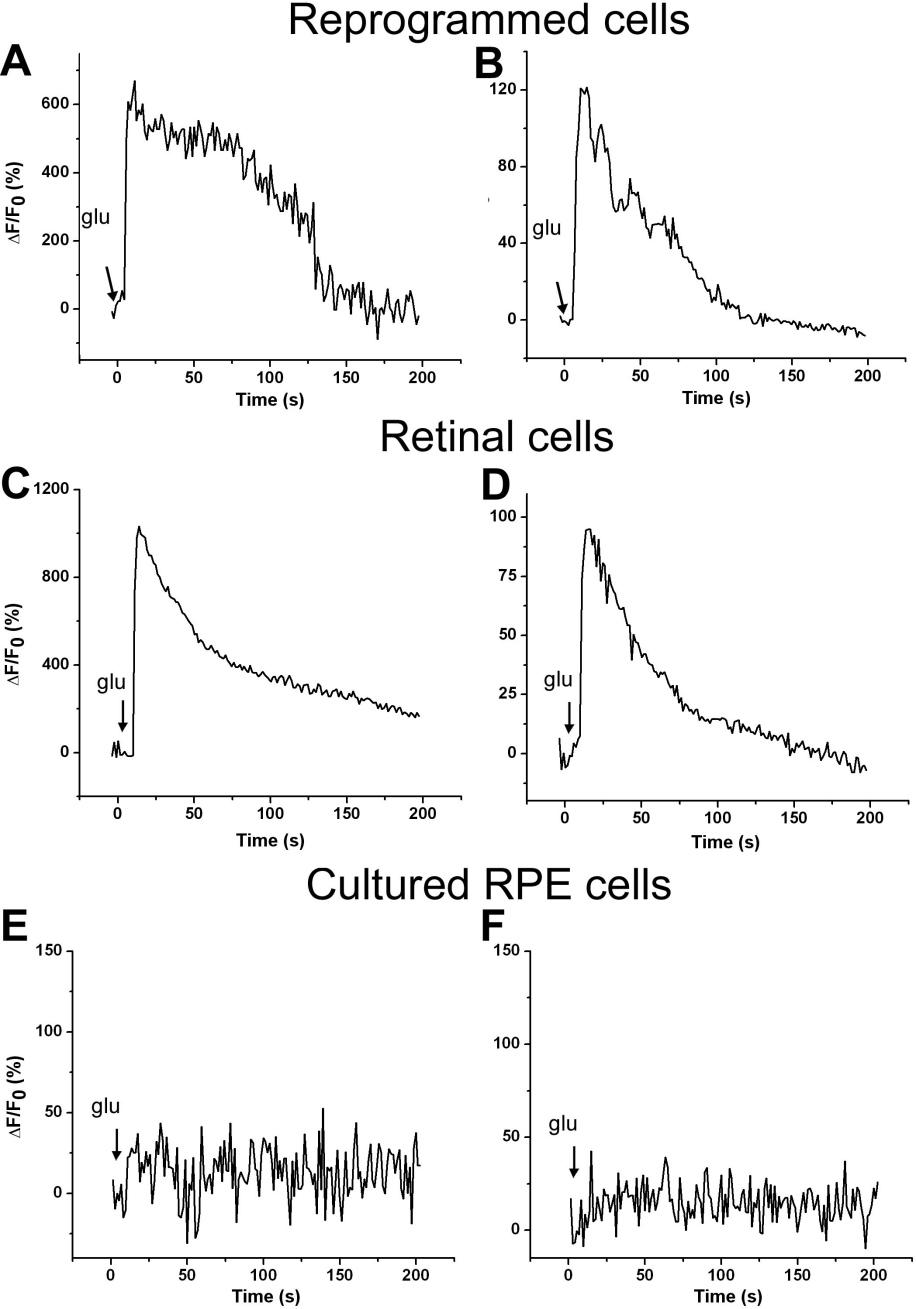
Reprogrammed cells showed responses to exogenous glutamate. **A, B:** The ΔF/Fo of each of two reprogrammed cells in an retinal pigment epithelium (RPE) cell culture infected with Replication Competent Avian Splice (RCAS)-*achaete-scute homolog 1* (*ash1)* was plotted against time (in seconds) to produce response curves. **C, D:** The ΔF/Fo of each of two retinal cells in an E16 chick retinal cell culture was plotted against time (in seconds) to produce response curves as reference to those of reprogrammed cells. **E, F:** The ΔF/Fo of each of two RPE cells in the control culture infected with RCAS was plotted against time to produce response curves as negative controls. While no responses were observed with RPE cells, reprogrammed cells responded to glutamate and the response curves were similar to that of retinal cells. The arrow in each plot points to the time at which the neurotransmitter was applied.

Reprogrammed cells also responded to 1 mM exogenous glutamate (Figure 6A,B). The response curves of the reprogrammed cells were similar to those of retinal cells. Like responses to NDMA+glycine, the extent of responses to glutamate varied among the cells. Varied responses were also observed with retinal neurons (Figure 6C,D). No similar responses were detected with cells in the control culture infected with RCAS (Figure 6E,F).

## Discussion

In this study, we tested *ash1* for its ability to induce de novo genesis of retinal neuron-like cells from RPE cell cultures. The first sign of *ash1*-induced changes in RPE progeny cells was the altered appearance of the culture, in which the presumptive RPE cells were unable to regain pigmentation, and clusters of neuron-like cells emerged in the otherwise cobblestone-like arrangement. *Ash1*-induced reprogramming was evidenced by the development of elaborate cellular processes. Fluorescence microscopy after labeling the cells with fluo-4 AM or with AAV-GFP DNA transfection illustrated that the reprogrammed cells formed multiple processes, which are typical of horizontal, amacrine, or ganglion cells, but not photoreceptors or bipolar cells.

Immunostaining and in situ hybridization analyses showed the expression of genes/markers that identify different types of retinal neurons. The most prevalently expressed marker was calretinin, suggesting that a major product of *ash1*-induced reprogramming might be cells that resembled amacrine, horizontal, as well as ganglion cells. Ganglion-like cells, nonetheless, were unlikely to be the major product, because of the relatively moderate number of RA4^+^ cells. Photoreceptor-like cells were unlikely to be the major product either, because most of the reprogrammed cells exhibited multiple processes, and a relatively moderate number of cells were IRBP^+^ and a very small number of cells were red opsin^+^. There were a large number of visinin^+^ cells in the *ash1*-reprogrammed RPE cell culture. However, the majority of them coexpressed calretinin, which indicates that, like in the retina where developing amacrine cells express photoreceptor-specific genes visinin [55,56,] and RxRγ [64], those amacrine-like cells also transiently expressed visinin. The absence of expression of bipolar marker *ath3* and *chx10* does not necessarily preclude the presence of bipolar-like cells in the reprogrammed cultures, as not all of the cell type-specific markers observed with retinal neurons are expressed in reprogrammed cells under experimental conditions [19,65]. Additionally, even though there were tens of thousands of calretinin^+^ cells in *ash1*-induced reprogramming, no AP2α^+^ cells were observed. The partial gene expression may reflect partial cell differentiation due to microenvironment or to a lack of other factors necessary for advancing neuronal differentiation to maturation. Nonetheless, the morphologies of the reprogrammed cells were less indicative of differentiation toward bipolar-like cells than toward other secondary neurons. Thus, the results from both molecular analysis and morphological examination favor the scenario of *ash1* reprogramming RPE progeny cells to differentiate toward amacrine-like cells as a major product, along with photoreceptor-like, ganglion-like, and possibly horizontal-like cells as accompanying products. This scenario is consistent with the observation that in the chick retina *ash1* increases the amacrine cell population (data not shown).

Reprogrammed cells, especially those with multiple processes, responded to exogenous glutamate and the administration of NMDA+glycine by transiently increasing their intracellular Ca^2+^ concentrations. Their fluorescence intensity increased in the cell body and in processes seconds after administration of the neurotransmitters, suggesting a fast calcium influx through calcium channels upon activation of their receptors. After reaching its peak, the fluorescence intensity decreased quickly and eventually returned to the basal level, a profile closely resembling the response profiles of retinal neurons. This suggests the presence of functional, ionotropic glutamate receptors in the reprogrammed cells. Together, the evidence of neural differentiation at the molecular, morphological, and physiologic levels indicates that *ash1* can reprogram RPE progeny cells to differentiate as cells that resemble certain neurons of the retina.
